# Behavioural profiling of social and locomotor responses to repeated ketamine exposure in adult zebrafish

**DOI:** 10.1098/rsos.260257

**Published:** 2026-11-11

**Authors:** Matheus Gallas-Lopes, Daniela V. Müller, Thailana Stahlhofer-Buss, Leonardo M. Bastos, Sofia Z. Becker, Samara M. Bruck, Angelo Piato, Ana P. Herrmann

**Affiliations:** Universidade Federal do Rio Grande do Sul, Porto Alegre, RS, Brazil

**Keywords:** ketamine, N-methyl-D-aspartate receptor hypofunction, social withdrawal, hyperlocomotion, stereotyped swimming, schizophrenia-like phenotypes

## Abstract

N-methyl-D-aspartate receptor antagonists are used to model schizophrenia-related phenotypes, but behavioural responses to repeated ketamine exposure in zebrafish remain poorly characterized. Three independent experiments were conducted to evaluate the acute, repeated and sustained behavioural effects of ketamine in adult zebrafish. In Experiment I, fish received 10, 20 or 40 mg l^−1^ ketamine daily for 5 days and were tested in the social preference test (SPT) and open tank test (OTT) on days 1, 5 and 7, with re-exposure on day 7 after a 48 h washout. Experiments II and III assessed whether behavioural changes persisted following 5- or 14-day exposure, with testing 48 h after the final treatment. Ketamine reduced social interaction and increased locomotor activity in the SPT on all days and increased rotational behaviour in the OTT on days 1 and 5. Measured responses did not progressively increase across test sessions, and most outcomes did not differ from controls 48 h after the final exposure in Experiments II and III. The results characterize the acute, concentration-dependent behavioural profile of ketamine in adult zebrafish and define the temporal window over which it is detectable. These findings differ from rodent reports of sustained effects and motivate further investigation across species.

## 1. Introduction

Animal models that recapitulate schizophrenia-like behavioural phenotypes are essential for uncovering the disorder's mechanisms and advancing translational research. Among the pharmacological tools used in preclinical studies, non-competitive *N*-methyl-d-aspartate (NMDA) receptor antagonists, such as dizocilpine (MK-801), phencyclidine and ketamine, are widely employed to induce behavioural alterations that reflect core symptom domains of schizophrenia [1–5]. By transiently blocking NMDA receptor signalling, these compounds contribute to dopaminergic hyperactivity in subcortical regions and evoke transient behavioural changes that resemble the positive, negative and cognitive symptoms associated with the disorder [2,5–8].

Rodent models have played a central role in preclinical schizophrenia research. However, a substantial number of pharmacological candidates that showed promise in these models have failed in clinical trials, highlighting a critical gap in translational success [9,10]. These failures raise concerns about the external validity of such models, particularly their ability to predict complex human outcomes. In response, there is growing recognition of the need for alternative models that can enhance translational relevance and reduce species-specific biases. One such strategy involves testing conserved behavioural and pharmacological effects across diverse species [11–14]. Among non-rodent models, zebrafish (*Danio rerio*) have emerged as a valuable organism in neurobehavioural research owing to their well-characterized nervous system, evolutionary conservation of key neurotransmitter pathways and suitability for high-throughput experimental designs [15–17]. These features make zebrafish a promising platform for cross-species validation and for improving the predictive value of preclinical findings.

Previous work from our group demonstrated that the NMDA receptor antagonist MK-801 disrupts social behaviour and induces hyperlocomotion in adult zebrafish, behaviours that have been proposed as relevant to schizophrenia research in this species [18,19]. Compared with MK-801, the behavioural effects of ketamine in zebrafish are less consistently described. Although ketamine is widely used in preclinical models of schizophrenia, studies in zebrafish vary considerably in design and outcomes tested, with limited agreement on its impact across social, locomotor and exploratory domains [1].

The effects of ketamine on zebrafish behaviour have been most commonly examined after acute exposures [1,20–22], and far less is known about behavioural changes following repeated administration or about whether these effects persist once treatment has ended. In rodents, repeated exposure to NMDA receptor antagonists results in progressively heightened behavioural responses or long-lasting changes that remain after drug clearance [23–28]. These findings suggest that both the temporal dynamics and the cumulative impact of ketamine exposure are important factors in shaping behavioural outcomes. Whether the effects observed during ketamine exposure also increase with repeated treatment or remain detectable after treatment ends is therefore important for evaluating the use of zebrafish alongside rodents.

This study aimed to characterize the behavioural effects of ketamine in adult zebrafish across three experimental protocols. Experiment I examined how behavioural responses evolved over repeated exposures and test sessions. Experiment II evaluated whether ketamine-induced behavioural alterations persisted after a short-term (5-day) exposure followed by a 48 h washout period. Experiment III extended this investigation by assessing whether a longer-term (14-day) exposure regimen would lead to detectable effects after the same washout interval. Together, these protocols allowed for the systematic assessment of both short- and long-term consequences of ketamine exposure across different behavioural domains.

## 2. Material and methods

This study was conducted in accordance with ethical guidelines and approved by the institutional animal ethics committee of the Universidade Federal do Rio Grande do Sul (approval no. 35525/2018). The reporting of this study follows the Animal Research: Reporting of *in vivo* Experiments (ARRIVE) guidelines for animal research transparency and rigour [29]. A completed ARRIVE Essential 10 checklist is available at osf.io/yqv6a. The experimental protocols for all three experiments were pre-registered on the Open Science Framework, specifying hypotheses, dependent variables, conditions, analyses, outlier exclusion criteria and sample size calculation [30] (Experiment I: osf.io/r7xdy [31]; Experiment II: osf.io/kjf3r [32]; Experiment III: osf.io/vkcft [33]).

### 2.1. Animals, housing and husbandry

A total of 224 adult wild-type shortfin zebrafish, aged between three and four months, were used across three separate experiments: 128 animals in Experiment I and 48 animals each in Experiments II and III. Of these, 217 completed the full experimental protocol and behavioural assessments (Experiment I: 122; Experiment II: 48; Experiment III: 47)—missing data are owing to the death of seven fish during the course of the experiments (full details in §2.10). The final sample included 111 females and 106 males, with the following sex distribution: Experiment I, 62 females and 60 males; Experiment II, 25 females and 23 males; Experiment III, 24 females and 23 males. The study was not designed or powered to assess sex-specific effects. Therefore, data from males and females were pooled for the analyses. Animals were sourced from a commercial supplier (Delphis, Porto Alegre, RS, Brazil). All animals were maintained at the zebrafish facility of the Instituto de Ciências Básicas da Saúde (Universidade Federal do Rio Grande do Sul) under a controlled 14 : 10 h light/dark cycle (lights on at 07.00). Prior to the onset of experiments, fish were allowed to acclimatize to laboratory conditions for at least two weeks. They were group-housed in 16 l glass tanks (40 × 20 × 24 cm) enriched with small PVC tubes and kept at a maximum density of two animals per litre. Tanks were filled with dechlorinated tap water and equipped with continuous aeration and mechanical, biological and chemical filtration systems (Altamar, Jacareí, SP, Brazil). Water quality parameters were maintained within acceptable ranges for zebrafish: temperature at 27 ± 2°C, pH 6.8 ± 0.3 and conductivity between 500 and 800 µS cm^−1^. Fish were fed twice daily with a combination of *Artemia salina* and commercial flake food (Poytara, Araraquara, SP, Brazil). All fish were naive and had not undergone behavioural testing before the start of their respective experiments. During the experimental phase, animals were redistributed into smaller aquariums (18 × 18 × 18 cm) according to the treatment group. Each group was housed separately, and all tanks were kept under identical conditions within the same facility, including lighting, temperature and vertical level. Tanks were coded to maintain carer blinding regarding treatment allocation. There were two housing tanks for each treatment group in all experiments. Physical barriers were used to prevent visual contact between groups, both in housing aquariums and during exposure to treatments. At the conclusion of the experiments, animals were euthanized using hypothermic shock. This procedure involved immersion in water cooled to 2–4°C for at least 2 min after cessation of opercular movements, followed by decapitation to ensure death. Sex was confirmed post-mortem via examination of the gonads.

### 2.2. Drugs

Ketamine solutions were prepared by diluting a 100 mg ml^−1^ injectable formulation (Cetamin, Syntec, Tamboré, SP, Brazil) in water maintained under the same conditions as the housing tanks. Ketamine solutions were freshly prepared on each day of exposure. In Experiment I, concentrations of 10, 20 and 40 mg l^−1^ (approx. 42, 84 and 168 µM, respectively) were used. In Experiments II and III, only the 40 mg l^−1^ (approx. 168 µM) concentration was used. These concentrations were selected based on previous studies reported in the literature [1,21,22]. Fish were exposed to the treatment solutions by immersion in a beaker for 20 min. The 20 min immersion period was chosen following established protocols reported in the literature and pilot experiments conducted by our group using another NMDA receptor antagonist in zebrafish [1,18,21,22]. The vehicle of the injectable formulation we used is water for injection. Therefore, control animals were handled in the same way but immersed in ketamine-free water under identical conditions, serving as an appropriate vehicle-matched control.

### 2.3. Experimental design

This study included three independent experiments, each conducted with a separate set of animals. The design and timeline for each experiment are illustrated in figure 1. Animals were allocated to the experimental groups using block randomization, ensuring balance across presumed sex and home tank origin. Because zebrafish sex cannot be confirmed without gonadal inspection, group allocation was counterbalanced based on external sex characteristics at the time of assignment. To minimize potential biases, the order of behavioural testing was determined using block randomization, with the sequence of randomized blocks generated via random.org, taking into account the test apparatuses to ensure even distribution across treatment conditions. The experimental unit in all analyses was a single animal, with each fish contributing one independent data point to the statistical comparisons. Both the experimenters conducting the behavioural assessments and those responsible for transferring the animals to the test apparatus were blinded to group allocation.

**Figure 1.**
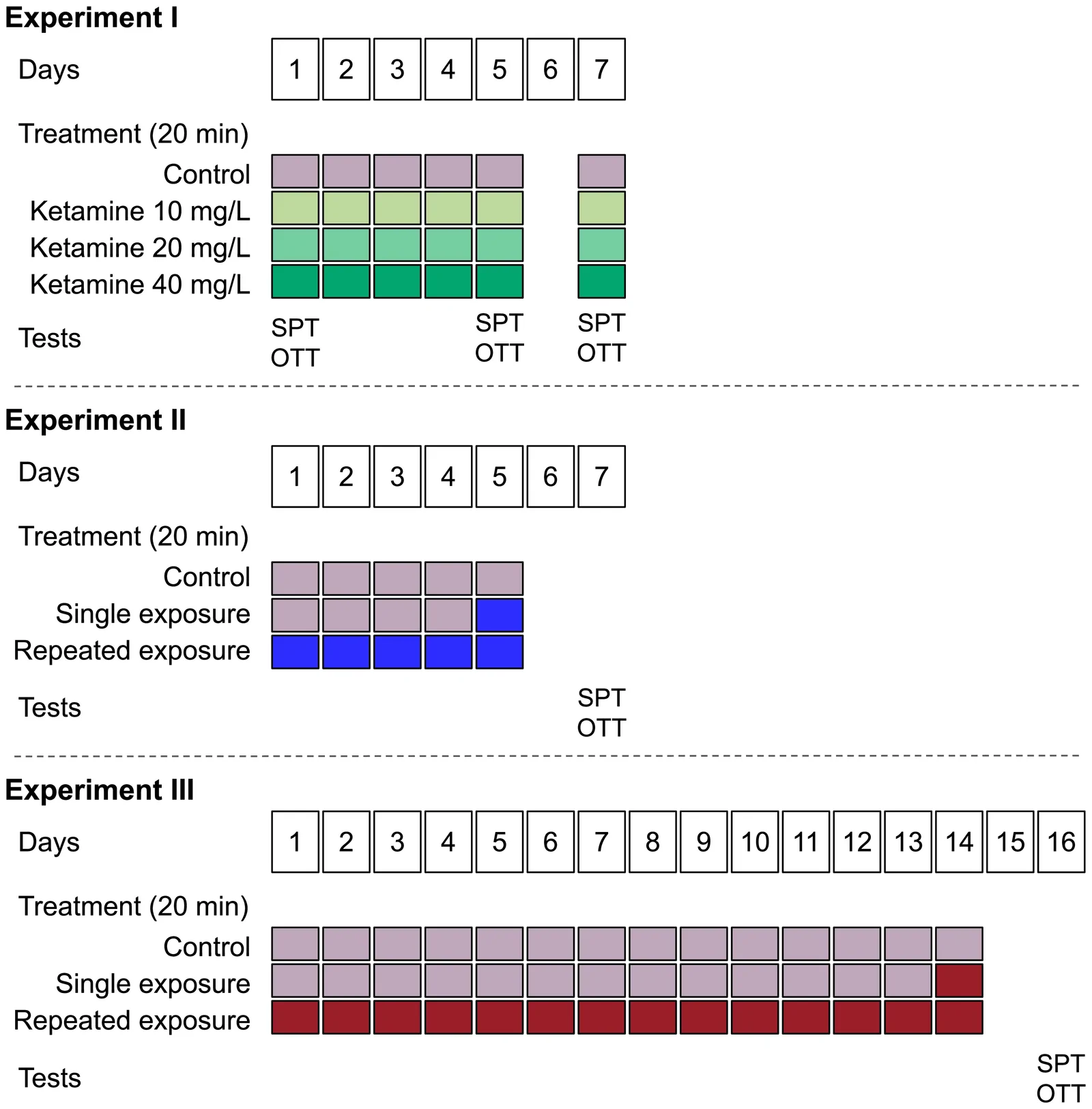
Overview of the experimental design evaluating the behavioural effects of ketamine exposure in zebrafish. Experiment I: fish were exposed via immersion to water or ketamine (10, 20 or 40 mg l^−1^) for 20 min daily over 5 days, with re-exposure on day 7. Behavioural tests (SPT, OTT) were conducted on days 1, 5 and 7. Experiment II: fish were exposed to water, a single ketamine immersion (40 mg l^−1^ on day 5) or repeated immersions (days 1–5). Tests were conducted on day 7. Experiment III: fish were exposed to water, a single immersion (day 14) or repeated daily immersions (days 1–14) of 40 mg l^−1^ ketamine. Testing occurred on day 16. SPT = social preference test; OTT = open tank test.

### 2.4. Experiment I

This experiment investigated the behavioural effects of repeated ketamine exposure at different concentrations. A total of 128 adult zebrafish were randomly assigned to one of four treatment groups (*n* = 32): control (17 females and 15 males), ketamine 10 mg l^−1^ (16 females and 16 males), ketamine 20 mg l^−1^ (15 females and 17 males) and ketamine 40 mg l^−1^ (16 females and 16 males). Missing data in the analysis reflect animal mortality as detailed in §2.10. Animals received a 20 min exposure to their assigned treatment once per day from experimental days 1 to 5. For each session, fish were transferred in groups of four to beakers containing 400 ml of the appropriate solution. After the washout period on day 6 (no handling or exposure), a final treatment was administered on day 7. This administration schedule was informed by prior research in mice, which demonstrated behavioural sensitization following 5 consecutive days of ketamine exposure and a subsequent 48 h washout period [27]. Following each exposure, animals were returned to their respective home tanks. Behavioural testing included both the social preference test (SPT) and the open tank test (OTT), conducted in sequence on days 1, 5 and 7 immediately after drug exposure. This experiment was designed to test the hypothesis that repeated exposure to ketamine reduces social interaction (as measured by time spent in the interaction zone during the SPT) and increases rotations in the OTT.

### 2.5. Experiment II

Building on the findings from Experiment I, the 40 mg l^−1^ ketamine concentration was selected for further investigation in Experiment II. This experiment evaluated whether behavioural changes induced by ketamine persist after treatment ends, comparing the effects of a single exposure to those of repeated administration. A total of 48 adult zebrafish were randomly assigned to one of three treatment groups (*n* = 16): control (11 females and 5 males), single exposure (7 females and 9 males) and repeated exposure (7 females and 9 males). Animals in the repeated group received a 20 min exposure to 40 mg l^−1^ ketamine once daily from experimental days 1 to 5. The single exposure group was treated with water from days 1 to 4 and exposed to 40 mg l^−1^ ketamine only on day 5. Control animals were exposed to water throughout the same period. For each exposure session, fish were transferred in groups of four to beakers containing 400 ml of the assigned solution. On day 6, animals remained in their home tanks without intervention. Behavioural testing was carried out on day 7, 48 h after the final exposure. All animals were evaluated in both the SPT and the OTT, in that order. This experiment was designed to test the hypothesis that repeated ketamine exposure induces sustained behavioural alterations as compared with a single exposure.

### 2.6. Experiment III

Following the results of Experiment II, a new experiment was designed to assess whether a longer exposure period would lead to sustained behavioural effects. A total of 48 adult zebrafish were randomly assigned to one of three treatment groups (*n* = 16): control (9 females and 7 males), single exposure (5 females and 11 males) and repeated exposure (10 females and 6 males). Missing data in the analysis reflect animal mortality as detailed in §2.10. Animals in the repeated group received a 20 min exposure to 40 mg l^−1^ ketamine once daily from experimental days 1 to 14. The single exposure group was treated with water from days 1 to 13 and received a single 40 mg l^−1^ ketamine exposure on day 14. Control animals were exposed to water throughout the same period. For each exposure session, fish were transferred in groups of four to beakers containing 400 ml of the assigned solution. On day 15, animals remained in their home tanks without intervention. Behavioural testing was carried out on day 16, 48 h after the final exposure. All animals were evaluated in both the SPT and the OTT, in that order. This experiment was designed to test the hypothesis that repeated ketamine exposure induces sustained behavioural alterations as compared with a single exposure.

### 2.7. Behavioural tests

The SPT and OTT were conducted sequentially to assess behavioural outcomes. Testing took place during the light phase, between 7.00 and 13.00. Animals were recorded from the front view during the SPT and from above during the OTT. Behavioural data were analysed using ANY-maze® tracking software (v. 7.20; Wood Dale, IL, USA). All researchers involved in analysing the recordings were blinded to group allocation.

### 2.8. Social preference test

The SPT was used to assess social affiliation in zebrafish [18,19]. After treatment, each animal was individually placed in a rectangular tank (30 × 10 × 15 cm) positioned between two adjacent tanks (15 × 10 × 13 cm each). One side tank contained a group of 10 conspecifics (social stimulus), while the other was left empty (neutral stimulus). A total of 40 stimulus fish, maintained in four groups of 10 animals, were used throughout the study. All tanks were filled with water under housing conditions to a height of 10 cm. The location of the social stimulus (left or right) was alternated across subjects to control for side preference. Water in the set-up was replaced between tests. For analysis, the test tank was virtually divided lengthwise into three equal zones: the interaction zone (nearest to the social stimulus), the neutral zone (nearest to the empty tank) and a central intermediate zone. Video recordings lasted 7 min, consisting of a 2 min habituation period followed by 5 min of data collection. The primary outcome was time spent in the interaction zone. Additional variables included total distance travelled, maximum speed, number of line crossings, percentage of distance travelled in the interaction zone and time spent immobile.

### 2.9. Open tank test

The OTT was conducted immediately after the SPT to evaluate locomotor activity and stereotyped swimming behaviour [18]. Each fish was placed in the centre of a circular white arena (24 cm in diameter, 8 cm high) filled with 2 cm of water under standard conditions. Recordings were captured from an overhead view for a 5 min session. The arena was refilled with clean water between trials. For behavioural analysis, the arena was virtually divided into two zones: a centre zone (12 cm in diameter) and a peripheral zone. The primary variable of interest was the number of rotations, which served as a marker of repetitive or stereotyped movement. Additional outcomes included total distance travelled, maximum speed, time spent in the centre zone, percentage of distance travelled in the centre, absolute turn angle and time spent immobile.

### 2.10. Statistical analysis

For Experiment I, the planned sample size was 48 animals per group (total = 192), based on a power of 0.88 to detect a medium effect size (*f* = 0.3, *α* = 0.05). After completing two batches (64 animals each), testing was halted owing to consistent results and effect sizes greater than expected. The final sample size was 32 per group (total = 128). A sensitivity analysis indicated that, for post hoc comparisons between two groups (*n* = 32 each), the design had sufficient power (1 − *β* = 0.88, *α* = 0.05) to detect a minimum effect size of *d* = 0.79. Since most of the observed effect sizes exceeded this threshold, the study was adequately powered to detect the effects identified in pairwise comparisons. No outliers were excluded. Six animals died during the experiment: three from the 40 mg l^−1^ group (two females during exposure on day 3; one male 1 h after exposure on day 4), one male in the control group (bitten by a tankmate on day 5) and one male from each of the 10 and 20 mg l^−1^ groups (after testing on day 5). For Experiments II and III, the initial plan was 32 animals per group (total = 96), based on a power of 0.94 to detect a large effect (*f* = 0.4, *α* = 0.05). After one batch (48 animals), testing was discontinued owing to the absence of clear treatment effects and in order to minimize animal use. The final sample size was 16 per group. One male from the control group died during Experiment III; no deaths occurred in Experiment II. Sample sizes were estimated using G*Power (v. 3.1; Düsseldorf, Germany). Effect sizes (Cohen's *d*) and 95% confidence intervals were calculated using R (v. 4.3.1), RStudio (v. 2023.12.1+402; Boston, MA, USA) and the meta package (v. 8.0-1) [34]. In Experiment I, exploratory subgroup analyses across test days were conducted using a random-effects model. Behavioural data were analysed in GraphPad Prism (v. 10.1.0; Boston, MA, USA). One-way ANOVA followed by Bonferroni post hoc tests was used for parametric outcomes; immobility time was analysed using the Kruskal–Wallis test followed by Dunn's correction. Results are reported as mean ± s.d. for normally distributed outcomes and as median with interquartile range for non-parametric data (as determined by inspecting the Q–Q plots). A *p*-value below 0.05 was considered statistically significant. Exploratory analyses of treatment, sex and their interaction were conducted for Experiment I on day 7 and for Experiments II and III, and results are available at osf.io/m4rbn.

### 2.11. Deviations from registered protocols

In Experiment I, the pre-registered sample size was 48 animals per group (total = 192), but data collection was stopped after 32 animals per group owing to consistent results and larger-than-expected effects. The planned analysis involved a two-way ANOVA with treatment group and experimental day as factors. However, because individual animals could not be reliably tracked across days, each day was analysed independently using one-way ANOVA to avoid inflating the sample size. Additionally, ‘per cent distance in the interaction zone’ and ‘per cent distance in the centre’ were not pre-registered outcomes but were included post hoc in Experiment I for consistency with Experiments II and III. In Experiments II and III, although 32 animals per group were planned, testing was halted after one batch (16 per group) owing to a lack of apparent treatment effects and to reduce unnecessary animal use, in accordance with the replacement, reduction and refinement (3Rs) principle [35]. Across all experiments, immobility time was analysed using Kruskal–Wallis followed by Dunn's test instead of ANOVA, as this outcome violated parametric assumptions.

## 3. Results

### 3.1. Experiment I

Ketamine administration led to consistent, concentration-dependent behavioural changes across tests. In the SPT, all concentrations reduced time spent in the interaction zone and per cent distance in the interaction zone, indicating decreased social preference. At the same time, ketamine increased total distance travelled, maximum speed and number of line crossings, reflecting a pattern of hyperlocomotion. In the OTT, ketamine also increased number of rotations, distance travelled and maximum speed, especially at higher concentrations, while the lower concentration increased time spent in the centre and per cent distance in the centre, an anxiolytic-like response typically interpreted as reduced thigmotaxis in an unfamiliar environment. Subgroup analyses revealed no significant differences across testing days for any outcome, showing no detectable progressive increase in the measured responses across test days. The following sections provide a more detailed account of these results.

#### 3.1.1. Social preference test

Figure 2 shows the effects of different ketamine concentrations (10, 20 and 40 mg l^−1^) in the SPT across three test sessions (days 1, 5 and 7). A significant effect of treatment was observed on each test day for the primary outcome, with ketamine reducing time spent in the interaction zone (day 1: *F*(3124)  =  32.96, *p* < 0.0001; day 5: *F*(3120) = 13.80, *p* < 0.0001; day 7: *F*(3118) = 12.73, *p* < 0.0001; figure 2A). Bonferroni post hoc comparisons showed that all ketamine concentrations significantly reduced social preference compared with the control group on all three days. Effect sizes are presented in figure 2B. On day 1, effect sizes were directly proportional to ketamine concentration, indicating a concentration-dependent increase in effect magnitude: −1.21 (95% CI [−1.75, −0.68]) for 10 mg l^−1^, −1.76 [−2.33, −1.18] for 20 mg l^−1^ and −2.87 [−3.57, −2.16] for 40 mg l^−1^. This pattern persisted on day 5 (−0.57 [−1.07, −0.06] for 10 mg l^−1^; −0.90 [−1.42, −0.38] for 20 mg l^−1^; −1.71 [−2.31, −1.12] for 40 mg l^−1^) and day 7 (−0.76 [−1.27, −0.24] for 10 mg l^−1^; −0.84 [−1.36, −0.32] for 20 mg l^−1^; −1.71 [−2.31, −1.12] for 40 mg l^−1^). No significant differences were found across testing days in the subgroup analysis (*p* =  0.2760).

**Figure 2.**
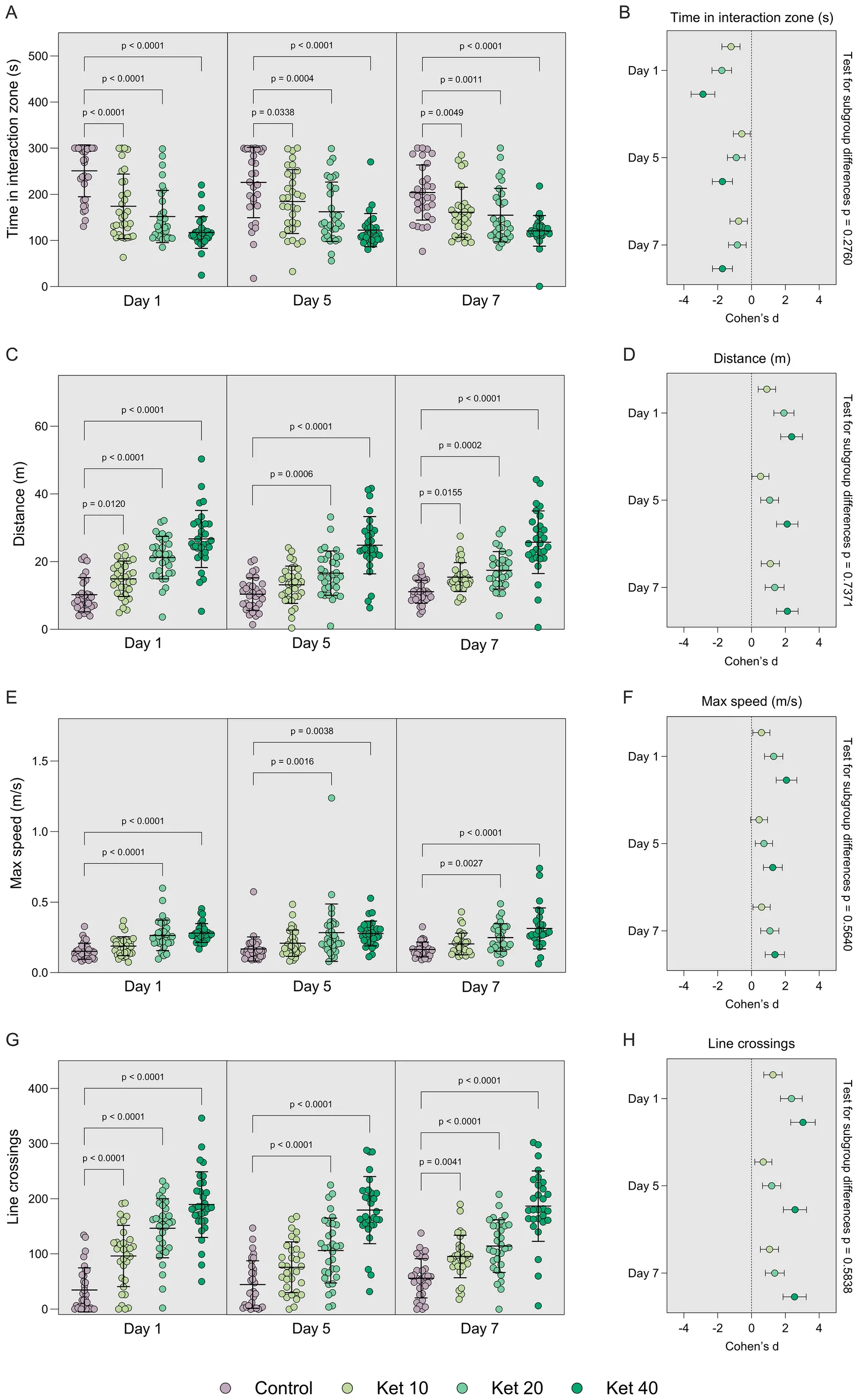
Effects of exposure to different concentrations of ketamine on the behaviour of adult zebrafish in the social preference test. (A) Time spent in the interaction zone, (C) total distance travelled, (E) maximum speed and (G) number of line crossings, measured on days 1, 5 and 7. (B, D, F and H) Corresponding effect sizes (Cohen's *d*) for each outcome, with test for subgroup differences across assessment days. One-way ANOVA followed by Bonferroni post hoc test. Data are presented as mean ± s.d. Effect sizes are expressed as Cohen's *d* with 95% confidence intervals. Subgroup differences tested using a random-effects model. Sample sizes: day 1: control = 32, ket 10 = 32, ket 20 = 32, ket 40 = 32; day 5:– control = 31, ket 10 = 32, ket 20 = 32, ket 40 = 29; day 7: control = 31, ket 10 = 31, ket 20 = 31, ket 40 = 29. Ket 10 = ketamine 10 mg l^−1^; ket 20 = ketamine 20 mg l^−1^; ket 40 = ketamine 40 mg l^−1^.

Ketamine also significantly increased total distance travelled during the test sessions (figure 2C). An effect of treatment was detected on all days (day 1: *F*(3124) = 40.58, *p* < 0.0001; day 5: *F*(3120) = 28.25, *p* < 0.0001; day 7: *F*(3118) = 31.25, *p* < 0.0001). Post hoc tests indicated significant increases at all concentrations on day 1, at 20 and 40 mg l^−1^ on day 5 and again for all groups on day 7. Effect sizes are shown in figure 2D. On each day, effect sizes increased with concentration: 0.91 [0.40, 1.43] for 10 mg l^−1^, 1.92 [1.32, 2.51] for 20 mg l^−1^ and 2.37 [1.73, 3.02] for 40 mg l^−1^ on day 1; 0.53 [0.03, 1.04] for 10 mg l^−1^, 1.08 [0.55, 1.61] for 20 mg l^−1^ and 2.12 [1.48, 2.75] for 40 mg l^−1^ on day 5; and 1.11 [0.58, 1.65] for 10 mg l^−1^, 1.37 [0.82, 1.93] for 20 mg l^−1^ and 2.12 [1.49, 2.76] for 40 mg l^−1^ on day 7. No significant differences were found across testing days in the subgroup analysis (*p* = 0.7371).

A significant main effect of treatment was also observed for maximum swimming speed (figure 2E), with increases detected on each test day (day 1: *F*(3124) = 20.31, *p* < 0.0001; day 5: *F*(3120) = 5.82, *p* = 0.001; day 7: *F*(3118) = 12.81, *p* < 0.0001). Post hoc analysis showed that only the 20 and 40 mg l^−1^ groups significantly differed from controls across all days, with no significant effects at 10 mg l^−1^. As shown in figure 2F, effect sizes increased with concentration: 0.59 [0.09, 1.09] for 10 mg l^−1^, 1.31 [0.77, 1.85] for 20 mg l^−1^ and 2.07 [1.46, 2.68] for 40 mg l^−1^ on day 1; 0.46 [−0.04, 0.96] for 10 mg l^−1^, 0.74 [0.22, 1.25] for 20 mg l^−1^ and 1.26 [0.71, 1.82] for 40 mg l^−1^ on day 5; and 0.60 [0.09, 1.11] for 10 mg l^−1^, 1.09 [0.55, 1.62] for 20 mg l^−1^ and 1.38 [0.82, 1.95] for 40 mg l^−1^ on day 7. No significant differences were found across testing days in the subgroup analysis (*p* = 0.5640).

Line crossings, another index of locomotor activity, were also increased following ketamine exposure (figure 2G) on all days (day 1: *F*(3124) = 51.21, *p* < 0.0001; day 5: *F*(3120) = 35.98, *p* < 0.0001; day 7: *F*(3118) = 39.69, *p* < 0.0001). All ketamine groups differed from controls on days 1 and 7, while only the 20 and 40 mg l^−1^ concentrations were significant on day 5. Effect sizes are shown in figure 2H. On each test day, values increased with concentration: 1.27 [0.73, 1.81] for 10 mg l^−1^, 2.37 [1.72, 3.01] for 20 mg l^−1^ and 3.05 [2.32, 3.77] for 40 mg l^−1^ on day 1; 0.70 [0.19, 1.21] for 10 mg l^−1^, 1.20 [0.66, 1.73] for 20 mg l^−1^ and 2.57 [1.88, 3.27] for 40 mg l^−1^ on day 5; and 1.06 [0.53, 1.60] for 10 mg l^−1^, 1.38 [0.83, 1.94] for 20 mg l^−1^ and 2.55 [1.86, 3.24] for 40 mg l^−1^ on day 7. No significant differences were found across testing days in the subgroup analysis (*p* = 0.5838).

Treatment also significantly decreased the per cent distance travelled within the interaction zone (supplementary material, figure S1A). An effect was observed on all test days (day 1: *F*(3124) = 28.64, *p* < 0.0001; day 5: *F*(3120) = 15.12, *p* < 0.0001; day 7: *F*(3118) = 7.67, *p* = 0.0001). Post hoc analysis confirmed that all concentrations significantly reduced this metric compared with controls on each day. Effect sizes are shown in figure S1B in the supplementary material. Effect magnitudes increased with concentration, reflected by more negative values: −1.13 [−1.66, −0.60] for 10 mg l^−1^, −1.67 [−2.24, −1.10] for 20 mg l^−1^ and −2.44 [−3.10, −1.79] for 40 mg l^−1^ on day 1; −0.75 [−1.26, −0.24] for 10 mg l^−1^, −1.10 [−1.64, −0.57] for 20 mg l^−1^ and −1.75 [−2.35, −1.15] for 40 mg l^−1^ on day 5; and −0.56 [−1.07, −0.06] for 10 mg l^−1^, −0.56 [−1.07, −0.06] for 20 mg l^−1^ and −1.22 [−1.77, −0.66] for 40 mg l^−1^ on day 7. No significant differences were found across testing days in the subgroup analysis (*p* = 0.0770).

No significant effect of ketamine was observed on time spent immobile during the SPT (supplementary material, figure S1C). Kruskal–Wallis tests yielded non-significant results on all test days (day 1: H = 7.09, *p* = 0.0692; day 5: *H* = 3.02, *p* = 0.3879; day 7: *H* = 1.77, *p* = 0.6223). Effect sizes are presented in figure S1D in the supplementary material. On day 1, values were −0.36 [−0.85, 0.14] for 10 mg l^−1^, −0.30 [−0.79, 0.19] for 20 mg l^−1^ and −0.17 [−0.66, 0.32] for 40 mg l^−1^. Day 5 values were 0.00 [−0.49, 0.50] for 10 mg l^−1^, −0.30 [−0.80, 0.19] for 20 mg l^−1^ and −0.43 [−0.95, 0.08] for 40 mg l^−1^. On day 7, effect sizes were −0.39 [−0.90, 0.11] for 10 mg l^−1^, −0.42 [−0.92, 0.08] for 20 mg l^−1^ and 0.15 [−0.36, 0.65] for 40 mg l^−1^. No significant differences were found across testing days in the subgroup analysis (*p* = 0.9385).

#### 3.1.2. Open tank test

Figure 3 summarizes the results of the OTT, assessing multiple behavioural outcomes in response to different ketamine concentrations (10, 20 and 40 mg l^−1^) across three sessions (days 1, 5 and 7). For the primary outcome, number of rotations, a significant effect of treatment was observed on day 1 (*F*(3124) = 5.32, *p* = 0.0018) and day 5 (*F*(3120) = 9.61, *p* < 0.0001), but not on day 7 (*F*(3118) = 1.73, *p* = 0.1637; figure 3). Post hoc comparisons indicated significant increases in rotations for animals treated with 20 and 40 mg l^−1^ on day 1 and for 40 mg l^−1^ on day 5. Effect sizes and 95% confidence intervals are shown in figure 3B. On day 1, values were as follows: 0.15 [−0.34, 0.64] for 10 mg l^−1^, 0.68 [0.18, 1.19] for 20 mg l^−1^ and 0.94 [0.42, 1.46] for 40 mg l^−1^. Day 5 showed a similar trend, with effect sizes of 0.22 [−0.27, 0.72] for 10 mg l^−1^, 0.38 [−0.12, 0.87] for 20 mg l^−1^ and 1.15 [0.60, 1.70] for 40 mg l^−1^. On day 7, effect sizes were 0.21 [−0.29, 0.71] for 10 mg l^−1^, −0.02 [−0.52, 0.47] for 20 mg l^−1^ and 0.63 [0.11, 1.15] for 40 mg l^−1^. Effect sizes increased with ketamine concentration across all days. No significant differences were found across testing days in the subgroup analysis (*p* = 0.4911).

**Figure 3.**
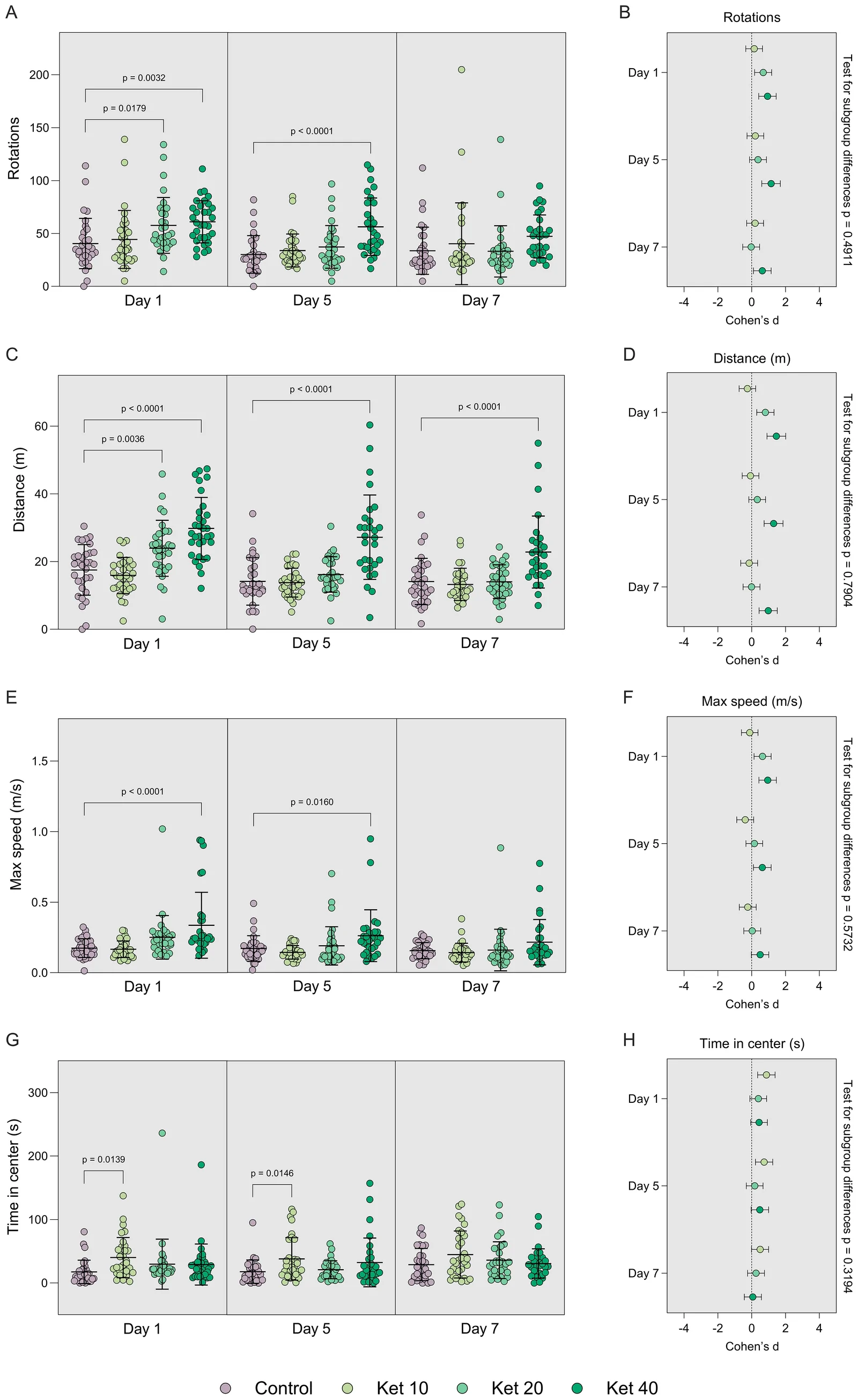
Effects of exposure to different concentrations of ketamine on the behaviour of adult zebrafish in the open tank test. (A) Number of rotations, (C) total distance travelled, (E) maximum speed and (G) time spent in the centre zone, measured on days 1, 5 and 7. (B, D, F and H) Corresponding effect sizes (Cohen's *d*) for each outcome, with test for subgroup differences across assessment days. One-way ANOVA followed by Bonferroni post hoc test. Data are presented as mean ± s.d. Effect sizes are expressed as Cohen's *d* with 95% confidence intervals. Subgroup differences tested using a random-effects model. Sample sizes: day 1: control = 32, ket 10 = 32, ket 20 = 32, ket 40 = 32; day 5: control = 31, ket 10 = 32, ket 20 = 32, ket 40 = 29; day 7: control = 31, ket 10 = 31, ket 20 = 31, ket 40 = 29. Ket 10 = ketamine 10 mg l^−1^; ket 20 = ketamine 20 mg l^−1^; ket 40 = ketamine 40 mg l^−1^.

For distance travelled, a significant effect of treatment was observed on day 1 (*F*(3124) = 21.81, *p* < 0.0001), day 5 (*F*(3120) = 19.72, *p* < 0.0001) and day 7 (*F*(3118) = 11.86, *p* < 0.0001; figure 3C). Post hoc comparisons revealed significant increases in distance for animals treated with 20 and 40 mg l^−1^ on day 1 and only 40 mg l^−1^ on days 5 and 7. Effect sizes and 95% confidence intervals are displayed in figure 3D. On day 1, values were −0.25 [−0.74, 0.24] for 10 mg l^−1^, 0.81 [0.30, 1.32] for 20 mg l^−1^ and 1.46 [0.91, 2.02] for 40 mg l^−1^. On day 5, effect sizes were −0.07 [−0.56, 0.43] for 10 mg l^−1^, 0.33 [−0.17, 0.83] for 20 mg l^−1^ and 1.30 [0.74, 1.86] for 40 mg l^−1^. On day 7, values were −0.14 [−0.64, 0.36] for 10 mg l^−1^, −0.01 [−0.50, 0.49] for 20 mg l^−1^ and 0.98 [0.44, 1.52] for 40 mg l^−1^. The magnitude of the effect sizes increased with ketamine concentration on each day. No significant differences were found across testing days in the subgroup analysis (*p* = 0.7904).

For maximum speed, a significant effect of treatment was found on day 1 (*F*(3124) = 9.34, *p* < 0.0001) and day 5 (*F*(3120) = 4.97, *p* = 0.0028), but not on day 7 (*F*(3118) = 2.31, *p* = 0.0799; figure 3E). Post hoc analyses revealed significant increases in maximum speed only for the 40 mg l^−1^ group on days 1 and 5. Effect sizes and 95% confidence intervals are presented in figure 3F. On day 1, values were −0.11 [−0.60, 0.38] for 10 mg l^−1^, 0.65 [0.14, 1.15] for 20 mg l^−1^ and 0.95 [0.43, 1.46] for 40 mg l^−1^. On day 5, values were −0.38 [−0.87, 0.12] for 10 mg l^−1^, 0.16 [−0.33, 0.66] for 20 mg l^−1^ and 0.63 [0.11, 1.15] for 40 mg l^−1^. On day 7, effect sizes were −0.23 [−0.73, 0.27] for 10 mg l^−1^, 0.04 [−0.46, 0.54] for 20 mg l^−1^ and 0.50 [−0.01, 1.02] for 40 mg l^−1^. No significant differences were found across testing days in the subgroup analysis (*p* = 0.5732).

For time spent in the centre, a significant effect of treatment was found on day 1 (*F*(3124) = 2.78, *p* = 0.0439) and day 5 (*F*(3120) = 3.65, *p* = 0.0146), but not on day 7 (*F*(3118) = 1.84, *p* = 0.1441; figure 3G). Post hoc comparisons indicated significantly increased centre time only for animals treated with 10 mg l^−1^ on days 1 and 5. Effect sizes and 95% confidence intervals are provided in figure 3H. On day 1, values were 0.87 [0.36, 1.39] for 10 mg l^−1^, 0.40 [−0.10, 0.89] for 20 mg l^−1^ and 0.44 [−0.06, 0.93] for 40 mg l^−1^. On day 5, effect sizes were 0.74 [0.23, 1.25] for 10 mg l^−1^, 0.18 [−0.31, 0.68] for 20 mg l^−1^ and 0.49 [−0.03, 1.00] for 40 mg l^−1^. By day 7, effects diminished: 0.50 [−0.01, 1.01] for 10 mg l^−1^, 0.26 [−0.24, 0.76] for 20 mg l^−1^ and 0.07 [−0.43, 0.58] for 40 mg l^−1^. The magnitude of the effect was highest for 10 mg l^−1^ across all days. Increased centre time is interpreted as an anxiolytic effect and was only observed at the lowest concentration. No significant differences were found across testing days in the subgroup analysis (*p* = 0.3194).

For per cent distance in the centre, a significant effect of treatment was detected on day 1 (*F*(3124) = 6.01, *p* = 0.0007) and day 5 (*F*(3120) = 3.18, *p* = 0.0266), but not on day 7 (*F*(3118) = 1.57, *p* = 0.2015; supplementary material, figure S2A). Post hoc analysis revealed significant increases only for animals treated with 10 mg l^−1^ on days 1 and 5. Effect sizes and 95% confidence intervals are shown in figure S2B in the supplementary material. On day 1, effect sizes were 1.03 [0.51, 1.55] for 10 mg l^−1^, 0.59 [0.09, 1.09] for 20 mg l^−1^ and 0.45 [−0.04, 0.95] for 40 mg l^−1^. Day 5 values were 0.71 [0.20, 1.22] for 10 mg l^−1^, 0.18 [−0.32, 0.67] for 20 mg l^−1^ and 0.43 [−0.08, 0.94] for 40 mg l^−1^. By day 7, effect sizes decreased: 0.44 [−0.06, 0.95] for 10 mg l^−1^, 0.25 [−0.25, 0.75] for 20 mg l^−1^ and 0.04 [−0.47, 0.55] for 40 mg l^−1^. Increased per cent distance in centre is interpreted as anxiolytic-like behaviour, observed only at the lowest ketamine concentration. No significant differences were found across testing days in the subgroup analysis (*p* = 0.1010).

For absolute turn angle, no significant effects of treatment were observed on day 1 (*F*(3124) = 0.62, *p* = 0.6036), day 5 (*F*(3120) = 1.11, *p* = 0.3485) or day 7 (*F*(3118) = 0.56, *p* = 0.6395; supplementary material, figure S2C). No group significantly differed from controls on any day. Effect sizes are shown in figure S2D in the supplementary material. These values were on day 1: −0.25 [−0.74, 0.24] for 10 mg l^−1^, 0.03 [−0.46, 0.52] for 20 mg l^−1^ and −0.07 [−0.56, 0.42] for 40 mg l^−1^; on day 5: −0.02 [−0.51, 0.48] for 10 mg l^−1^, −0.37 [−0.86, 0.13] for 20 mg l^−1^ and −0.15 [−0.66, 0.36] for 40 mg l^−1^; and on day 7: −0.08 [−0.58, 0.41] for 10 mg l^−1^, −0.27 [−0.77, 0.23] for 20 mg l^−1^ and −0.02 [−0.53, 0.48] for 40 mg l^−1^. No significant differences were found across testing days in the subgroup analysis (*p* = 0.8217).

No significant effect of ketamine was observed on time spent immobile during the OTT on any of the three experimental days (day 1: *H* = 7.68, *p* = 0.053; day 5: *H* = 6.73, *p* = 0.0812; day 7: *H* = 3.63, *p* = 0.3038; supplementary material, figure S2E). Effect sizes and 95% confidence intervals are shown in figure S2F in the supplementary material. These values were on day 1: −0.24 [−0.73, 0.25] for 10 mg l^−1^, −0.29 [−0.78, 0.21] for 20 mg l^−1^ and −0.36 [−0.86, 0.13] for 40 mg l^−1^; on day 5: −0.27 [−0.77, 0.22] for 10 mg l^−1^, −0.29 [−0.79, 0.21] for 20 mg l^−1^ and −0.48 [−1.00, 0.03] for 40 mg l^−1^; and on day 7: −0.43 [−0.93, 0.08] for 10 mg l^−1^, −0.30 [−0.80, 0.20] for 20 mg l^−1^ and −0.44 [−0.95, 0.07] for 40 mg l^−1^. No significant differences were found across testing days in the subgroup analysis (*p* = 0.2612).

### 3.2. Experiment II

Ketamine administration at 40 mg l^−1^ failed to produce sustained behavioural alterations following either acute or repeated exposure. In the SPT, neither a single administration on day 5 nor repeated treatment over 5 consecutive days affected time spent in the interaction zone, per cent distance in the interaction zone or locomotor-related measures such as distance travelled, line crossings, maximum speed or time immobile. Likewise, in the OTT, no significant effects were observed for number of rotations, distance travelled, time spent in the centre, per cent distance in the centre, absolute turn angle or time immobile. A significant reduction in maximum speed was detected only in the group receiving repeated administration, suggesting a limited and transient effect. Overall, the absence of robust behavioural changes after a 48 h washout indicates that neither acute nor repeated ketamine exposure at this concentration produced sustained effects. The following sections provide a more detailed account of these results.

#### 3.2.1. Social preference test

Figure 4 shows the effects of single and repeated ketamine exposure (40 mg l^−1^) on behaviour in the SPT. In this protocol, ketamine was administered daily for 5 consecutive days, and behavioural testing was conducted 48 h after the final concentration. For the primary outcome, time spent in the interaction zone, no significant effect of treatment was observed (*F*(2,45) = 0.40, *p* = 0.6701; figure 4A). Effect sizes are shown in figure 4G: −0.32 [−1.02, 0.37] for the single exposure group and −0.11 [−0.81, 0.58] for the repeated group. For distance travelled, there was also no significant treatment effect (*F*(2,45) = 0.60, *p* = 0.5524; figure 4B), with effect sizes of 0.25 [−0.45, 0.94] and 0.42 [−0.28, 1.12] for single and repeated exposures, respectively. No effect of treatment was detected for maximum speed (*F*(2,45) = 0.22, *p* = 0.8066; figure 4C), and effect sizes were 0.13 [−0.56, 0.83] for single and −0.10 [−0.79, 0.59] for repeated exposure. Similarly, no significant effect was found for the number of line crossings (*F*(2,45) = 0.09, *p* = 0.9137; figure 4D), with effect sizes of 0.11 [−0.59, 0.80] and −0.05 [−0.74, 0.65], respectively. For per cent distance in the interaction zone (*F*(2,45) = 0.09, *p* = 0.9172; figure 4E), results were non-significant, with effect sizes of −0.10 [−0.80, 0.59] for single and 0.05 [−0.65, 0.74] for repeated exposure. Finally, for time immobile, no significant difference was found (*H* = 0.325, *p* = 0.8501; figure 4F), and the effect sizes were 0.31 [−0.39, 1.01] for single and 0.12 [−0.58, 0.81] for repeated exposure.

**Figure 4.**
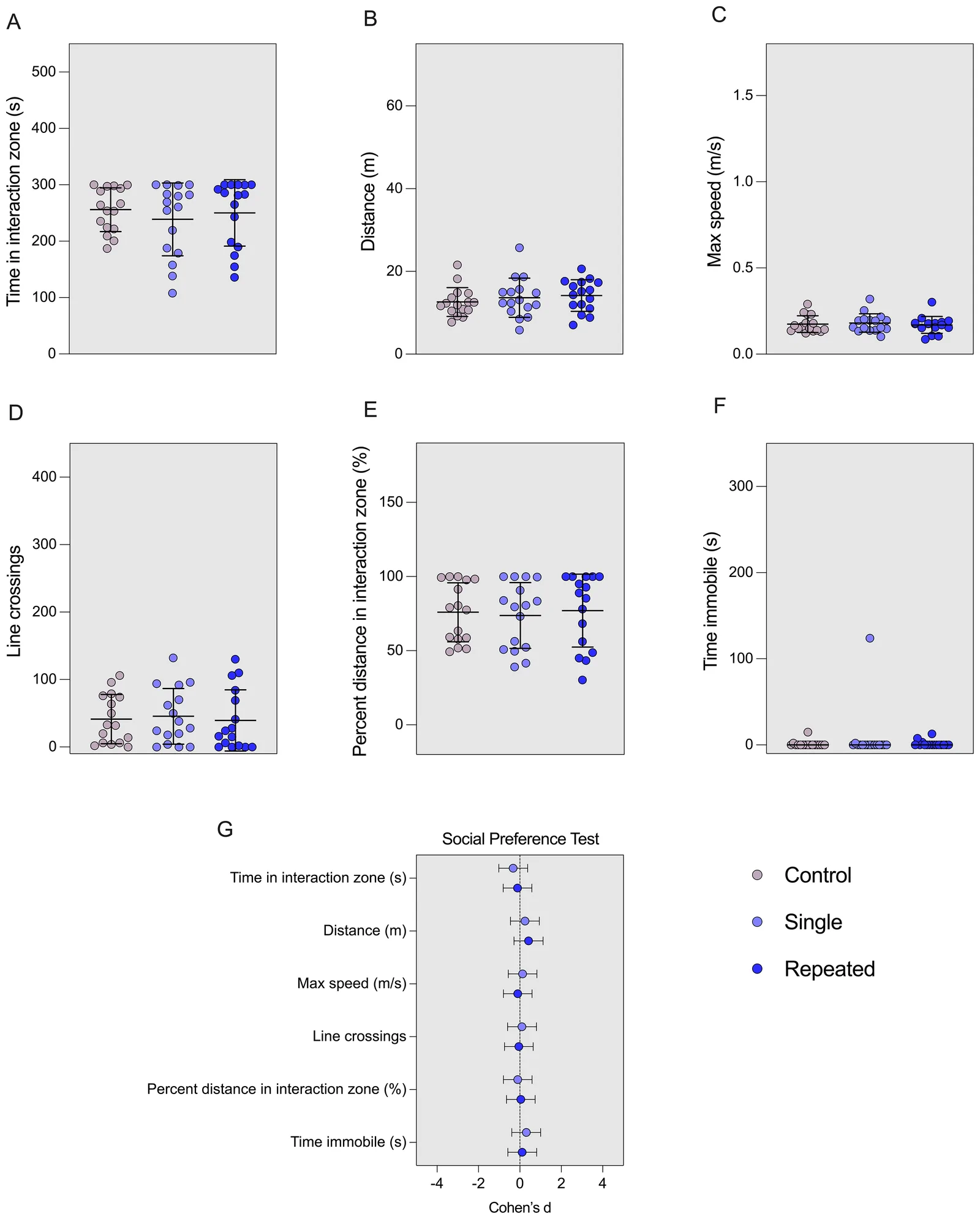
Effects of single (day 5) or repeated (days 1–5) ketamine exposures on the behaviour of adult zebrafish in the social preference test. (A) Time spent in the interaction zone, (B) total distance travelled, (C) maximum speed, (D) number of line crossings, (E) per cent of distance travelled in the interaction zone and (F) time spent immobile. (G) Effect sizes (Cohen's *d*) for the single and repeated exposure groups across the outcomes assessed. One-way ANOVA followed by Bonferroni post hoc test was used for A–E. Kruskal–Wallis test followed by Dunn's post hoc was used for F. Data are presented as mean ± s.d. for A–E and as median with interquartile range for F. Effect sizes are expressed as Cohen's *d* with 95% confidence intervals. Sample sizes: control = 16, single = 16, repeated = 16. Single = single ketamine exposure (40 mg l^−1^) on day 5; repeated = daily ketamine exposures (40 mg l^−1^) from day 1 to 5.

#### 3.2.2. Open tank test

Figure 5 presents the results of the OTT conducted after single or repeated ketamine exposure (40 mg l^−1^). In this protocol, ketamine was administered daily for 5 consecutive days, and behavioural testing was conducted 48 h after the final concentration. For the primary outcome, number of rotations, no significant differences were detected between groups (*F*(2,45) = 0.52, *p* = 0.5994; figure 5A), with effect sizes of 0.12 [−0.57, 0.82] and 0.33 [−0.37, 1.03] for the single and repeated groups, respectively. For distance travelled, results were not significant (*F*(2,45) = 0.36, *p* = 0.6991; figure 5B), and the effect sizes were −0.11 [−0.80, 0.58] for single and 0.18 [−0.51, 0.88] for repeated exposure. Maximum speed showed a significant treatment effect (*F*(2,45) = 3.61, *p* = 0.0352; figure 5C), and post hoc comparisons indicated a significant decrease in the repeated ketamine group relative to control. Effect sizes were −0.71 [−1.42, 0.01] for single and −0.82 [−1.55, −0.10] for repeated exposure. For time spent in the centre, no significant effect was found (*F*(2,45) = 1.51, *p* = 0.2316; figure 5D), and the corresponding effect sizes were 0.45 [−0.25, 1.16] and 0.13 [−0.56, 0.83]. Likewise, no treatment effect was observed for per cent distance in the centre (*F*(2,45) = 1.39, *p* = 0.2608; figure 5E), with effect sizes of 0.39 [−0.31, 1.09] and −0.23 [−0.92, 0.47], respectively. No differences were found for absolute turn angle (*F*(2,45) = 0.35, *p* = 0.7091; figure 5F), with effect sizes of 0.03 [−0.66, 0.73] and 0.29 [−0.41, 0.99]. Lastly, for time immobile, no significant difference was observed (*H* = 0.302, *p* = 0.8597; figure 5G), and effect sizes were 0.00 [−0.69, 0.69] and −0.36 [−1.06, 0.34] for single and repeated exposure, respectively.

**Figure 5.**
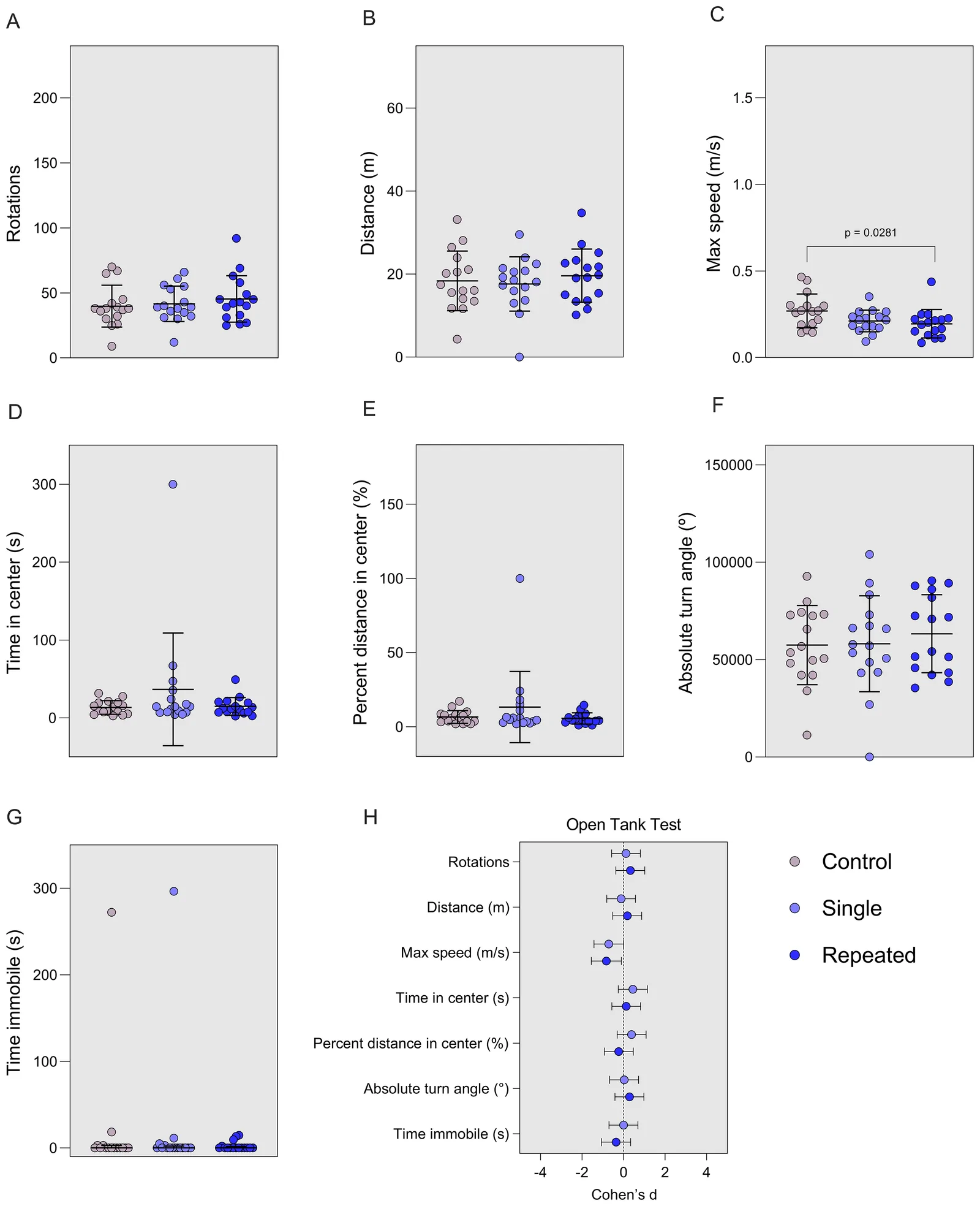
Effects of single (day 5) or repeated (days 1–5) ketamine exposures on the behaviour of adult zebrafish in the open tank test. (A) Number of rotations, (B) total distance travelled, (C) maximum speed, (D) time spent in the centre zone, (E) per cent of distance travelled in the centre zone, (F) absolute turn angle and (G) time spent immobile. (H) Effect sizes (Cohen's *d*) for the single and repeated exposure groups across the outcomes assessed. One-way ANOVA followed by Bonferroni post hoc test was used for A–F. Kruskal–Wallis test followed by Dunn's post hoc was used for G. Data are presented as mean ± s.d. for A–F and as median with interquartile range for G. Effect sizes are expressed as Cohen's *d* with 95% confidence intervals. Sample sizes: control = 16, single = 16, repeated = 16. Single = single ketamine exposure (40 mg l^−1^) on day 5; repeated = daily ketamine exposures (40 mg l^−1^) from day 1 to 5.

### 3.3. Experiment III

Ketamine administration at 40 mg l^−1^ did not produce sustained behavioural effects following either single exposure on day 14 or repeated administration over 14 consecutive days. In the SPT, no differences were observed in time spent in the interaction zone, distance travelled, maximum speed, line crossings or per cent distance in the interaction zone 48 h after treatment, with the exception of increased time immobile in the repeated group. In the OTT, no treatment effects were detected across any of the outcomes, including number of rotations, distance travelled, maximum speed, time in the centre, per cent distance in the centre, absolute turn angle and time immobile. Together, these findings indicate a lack of persistent behavioural alterations after ketamine exposure under this protocol. The following sections provide a more detailed account of these results.

#### 3.3.1. Social preference test

Figure 6 shows the effects of single and repeated ketamine exposure (40 mg l^−1^) on behaviour in the SPT. In this protocol, ketamine was administered daily for 14 consecutive days, and behavioural testing was conducted 48 h after the final concentration. For the primary outcome, time spent in the interaction zone, no significant effect of treatment was observed (*F*(2,44) = 1.56, *p* = 0.2214; figure 6A). Effect sizes were 0.48 [−0.23, 1.20] for the single exposure group and 0.54 [−0.17, 1.26] for the repeated group (figure 6G). For distance travelled, no significant differences were found (*F*(2,44) = 1.95, *p* = 0.1539; figure 6B), with effect sizes of −0.57 [−1.29, 0.15] and −0.59 [−1.31, 0.14] for the single and repeated groups, respectively. Similarly, no significant treatment effect was observed for maximum speed (*F*(2,44) = 0.10, *p* = 0.9049; figure 6C), with effect sizes of −0.12 [−0.83, 0.58] for single and 0.02 [−0.68, 0.73] for repeated exposure. Line crossings did not differ significantly across groups (*F*(2,44) = 1.86, *p* = 0.1678; figure 6D), with effect sizes of −0.53 [−1.25, 0.19] for single and −0.58 [−1.30, 0.14] for repeated treatment. For per cent distance in the interaction zone, results were non-significant (*F*(2,44) = 1.09, *p* = 0.3444; figure 6E), and effect sizes were 0.33 [−0.38, 1.04] and 0.50 [−0.22, 1.22], respectively. Finally, for time immobile, a significant treatment effect was observed (*H* = 6.788, *p* = 0.0336; figure 6F), with post hoc comparisons indicating significantly greater immobility in the repeated group compared with controls. The effect sizes were −0.01 [−0.71, 0.69] for single and 0.39 [−0.32, 1.11] for repeated ketamine exposure (figure 6G), suggesting an increase in immobility following chronic treatment.

**Figure 6.**
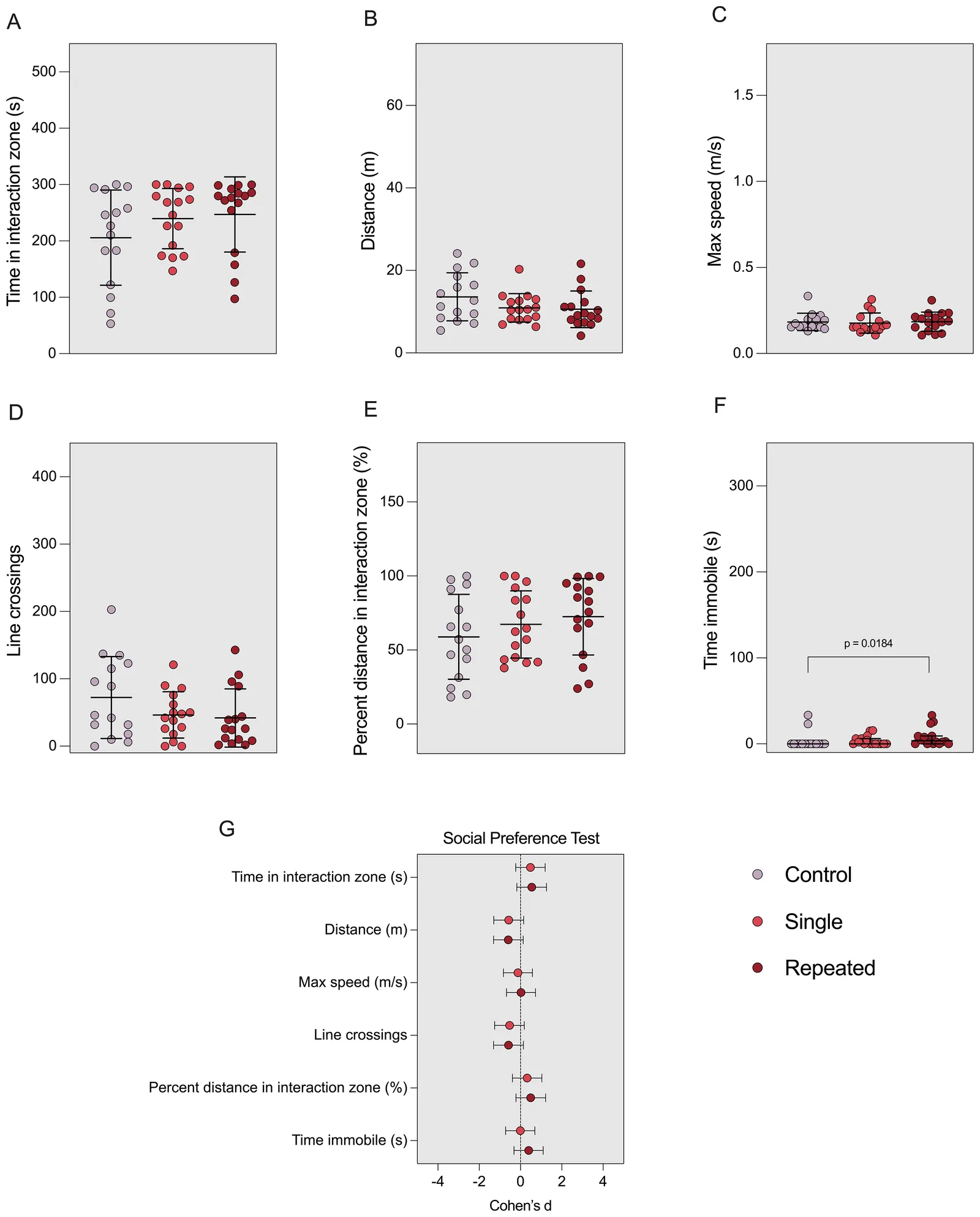
Effects of single (day 14) or repeated (days 1–14) ketamine exposures on the behaviour of adult zebrafish in the social preference test. (A) Time spent in the interaction zone, (B) total distance travelled, (C) maximum speed, (D) number of line crossings, (E) per cent of distance travelled in the interaction zone and (F) time spent immobile. (G) Effect sizes (Cohen's *d*) for the single and repeated exposure groups across the outcomes assessed. One-way ANOVA followed by Bonferroni post hoc test was used for A–E. Kruskal–Wallis test followed by Dunn's post hoc was used for F. Data are presented as mean ± s.d. for A–E and as median with interquartile range for F. Effect sizes are expressed as Cohen's *d* with 95% confidence intervals. Sample sizes: control = 15, single = 16, repeated = 16. Single = single ketamine exposure (40 mg l^−1^) on day 14; repeated = daily ketamine exposures (40 mg l^−1^) from day 1 to 14.

#### 3.3.2. Open tank test

Figure 7 presents the results of the OTT following single or repeated ketamine exposure (40 mg l^−1^) over 14 days, with testing conducted after a 48 h washout. For the primary outcome, number of rotations, no significant treatment effects were found (*F*(2,44) = 0.06, *p* = 0.9458; figure 7A), and effect sizes were 0.11 [−0.60, 0.81] and 0.05 [−0.66, 0.75] for single and repeated groups, respectively (figure 7H). No significant differences were observed for distance travelled (*F*(2,44) = 0.01, *p* = 0.9939; figure 7B), with effect sizes of 0.04 [−0.67, 0.74] and 0.02 [−0.68, 0.73]. Maximum speed also did not differ across groups (*F*(2,44) = 2.00, *p* = 0.1475; figure 7C), and effect sizes were −0.71 [−1.43, 0.02] for single and −0.49 [−1.21, 0.22] for repeated exposure. For time spent in the centre of the tank, no treatment effects were observed (*F*(2,44) = 0.09, *p* = 0.9146; figure 7D), and effect sizes were 0.14 [−0.57, 0.84] and 0.12 [−0.58, 0.83]. Per cent distance in the centre also showed no significant differences (*F*(2,44) = 0.62, *p* = 0.5407; figure 7E), with effect sizes of −0.25 [−0.96, 0.46] and −0.41 [−1.12, 0.30], respectively. No significant treatment effect was found for absolute turn angle (*F*(2,44) = 0.46, *p* = 0.6368; figure 7F), with effect sizes of 0.27 [−0.44, 0.98] and 0.33 [−0.38, 1.04]. Finally, time immobile did not differ significantly across groups (*H* = 2.585, *p* = 0.2746; figure 7G), and effect sizes were −0.45 [−1.16, 0.27] for single and −0.37 [−1.09, 0.34] for repeated exposure (figure 7H).

**Figure 7.**
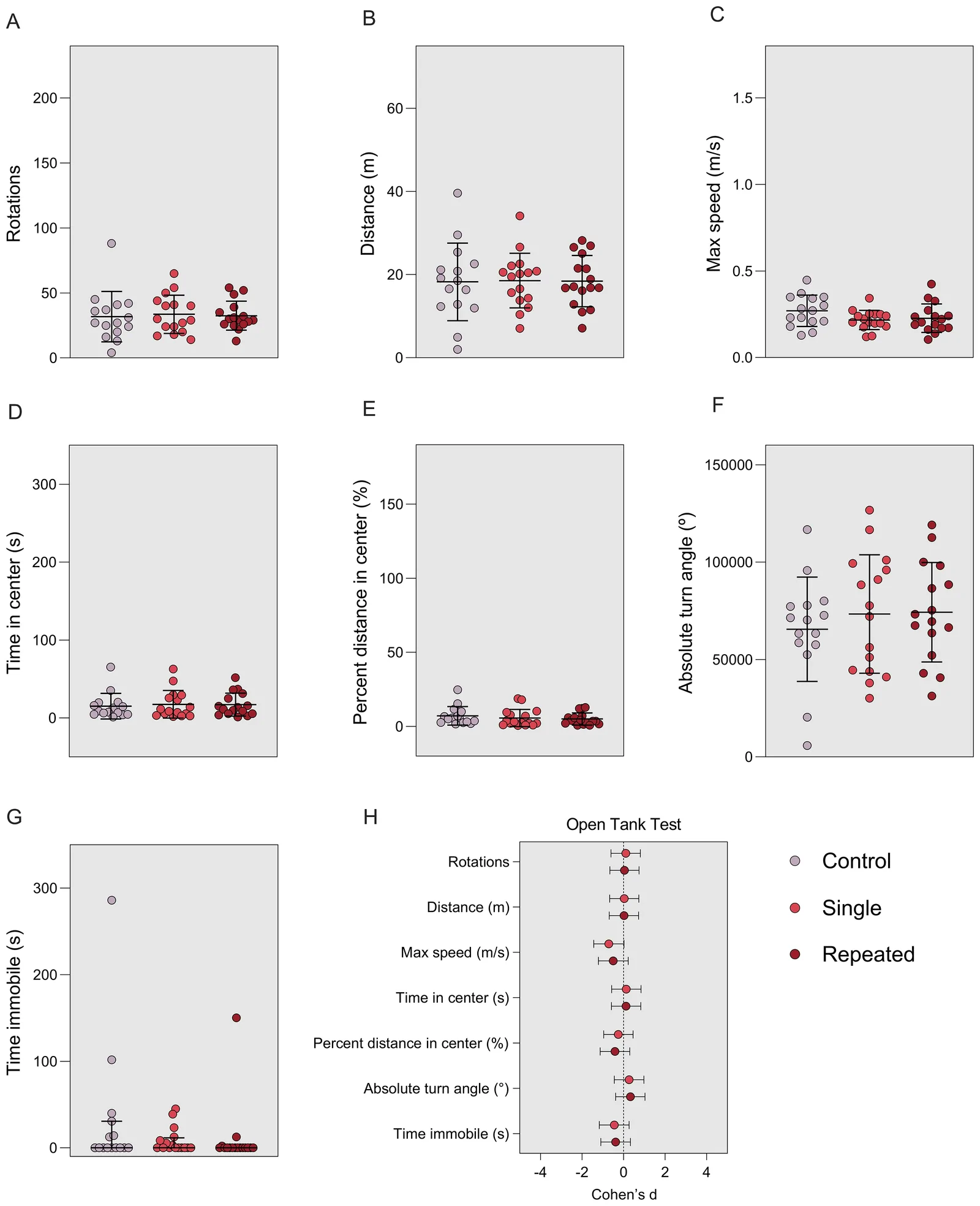
Effects of single (day 14) or repeated (days 1–14) ketamine exposures on the behaviour of adult zebrafish in the open tank test. (A) Number of rotations, (B) total distance travelled, (C) maximum speed, (D) time spent in the centre zone, (E) per cent of distance travelled in the centre zone, (F) absolute turn angle and (G) time spent immobile. (H) Effect sizes (Cohen's *d*) for the single and repeated exposure groups across the outcomes assessed. One-way ANOVA followed by Bonferroni post hoc test was used for A–F. Kruskal–Wallis test followed by Dunn's post hoc was used for G. Data are presented as mean ± s.d. for A–F and as median with interquartile range for G. Effect sizes are expressed as Cohen's *d* with 95% confidence intervals. Sample sizes: control = 15, single = 16, repeated = 16. Single = single ketamine exposure (40 mg l^−1^) on day 14; repeated = daily ketamine exposures (40 mg l^−1^) from day 1 to 14.

## 4. Discussion

Taken together, our data delineate the scope of repeated ketamine exposure in zebrafish as an NMDA receptor hypofunction paradigm, showing acute effects, no progressive increase in the measured responses across test sessions and no persistent effects under the schedules used here. Literature reviews highlight discrepancies regarding the effects of NMDA antagonists on social and locomotor parameters in zebrafish, often reflecting variation in compound type, dosing protocols and behavioural assays [1]. In our study, ketamine induced acute, concentration-dependent behavioural alterations in adult zebrafish that were robust and reproducible. In the SPT, ketamine reduced social interaction and increased locomotor activity across all concentrations, while in the OTT it led to more selective effects, including transient increases in rotational behaviour and exploratory movement. Behavioural responses did not progressively increase across test sessions, and ketamine-induced changes were not detected after a 48 h washout in the 5- and 14-day protocols. If anything, the OTT findings from Experiment I may point to a potential tolerance effect, as certain behaviours observed on day 1 diminished over subsequent experimental days. However, it remains unclear whether this reflects a true drug tolerance effect or merely results from repeated testing. These results suggest that, under the exposure conditions used here, ketamine's effects are reversible and restricted to the acute phase. Although no persistent alterations were observed, we cannot rule out the possibility that zebrafish might display chronic or progressive phenotypes under alternative experimental designs.

Experiment I revealed a clear, concentration-dependent profile of behavioural alterations following ketamine exposure. In the SPT, zebrafish showed a robust decrease in social preference across all concentrations, indicated by reduced time and per cent distance in the interaction zone. This phenotype parallels the social withdrawal modelled as a negative symptom in rodent studies and is consistent with effects of NMDA receptor antagonists in mammals [36–41]. Ketamine also induced hyperlocomotion, evidenced by increased distance travelled, swimming speed and zone crossings. Increased locomotor activity has been widely used as a behavioural read-out relevant to the positive symptoms of schizophrenia [2,42,43]. These behavioural changes are in line with findings from other NMDA antagonists in zebrafish, which similarly induce both social withdrawal and hyperlocomotion [18,19,44–47].

The OTT revealed a more selective profile. Rotational behaviour, often interpreted as a marker of stereotypy or repetitive movement, increased transiently at higher concentrations but was not sustained beyond early sessions. This has also been reported in previous studies, indicating a reproducible effect on repetitive behaviour [21,22,48]. Absolute turn angle remained unaffected, and ketamine's impact on general locomotor outcomes in the OTT was milder than in the SPT, emphasizing the influence of test context. Earlier work also reported ketamine-induced circling without increased general locomotion in zebrafish, consistent with the possibility that these behavioural outcomes vary across testing contexts [49]. Previous findings indicate that NMDA antagonist-induced hyperlocomotion is modulated by environmental novelty, with stronger effects typically observed in unfamiliar settings or in response to novel stimuli [18,46]. In our study, hyperlocomotion was greater in the SPT compared with the OTT, probably owing to the contextual salience of the task, where proximity to the conspecifics may enhance arousal or motivational drive. Since the OTT was conducted after the SPT, we cannot rule out the possibility that this difference reflects a testing order effect. However, prior MK-801 studies using separate cohorts also found hyperlocomotion in the SPT but not the OTT, supporting the higher locomotor sensitivity of the SPT [18]. Similarly, studies using ketamine in the novel tank test, a context involving only exposure to a new arena, have also failed to detect hyperlocomotion, further supporting the idea that this phenotype is highly dependent on the salience of the testing environment [22,50].

The lowest ketamine concentration elicited an anxiolytic-like response, reflected by greater centre time and distance in the OTT. Other studies using ketamine have reported anxiolytic-like effects, but at higher concentrations than those tested here (20–60 mg l^−1^), particularly in the novel tank and light–dark tests [20,22]. However, these findings require further characterization, as ketamine-induced changes in locomotor activity may confound anxiety-related outcomes. In rodents, ketamine has also been shown to produce anxiolytic- or antidepressant-like effects [51–53], though this may only emerge in animals exposed to stress protocols [54,55]. Whether a similar stress-dependent effect exists in zebrafish remains to be tested.

Unlike the study that inspired Experiment I [27], behavioural responses did not progressively increase across test days. Instead, responses remained mainly consistent, contrasting with rodent models where repeated NMDA receptor blockade produces progressively amplified behavioural effects [26,28,56–58]. This is in line with previous zebrafish research showing no evidence of tolerance or sensitization in circling behaviour following 5 days of ketamine exposure, suggesting a stable behavioural profile over repeated administrations [48]. A similar absence of cumulative behavioural effects across 7 days of MK-801 exposure has been reported in adult zebrafish [59].

Experiments II and III assessed behavioural persistence after 5- and 14-day exposure schedules, each followed by a 48 h washout. In both cases, zebrafish behaviour returned to baseline, with no lasting effects observed in any of the outcomes. Minor reductions in maximum speed after 5 days and increased immobility after 14 days were observed, indicating transient effects in adult zebrafish.

Studies examining the sustained effects of ketamine in zebrafish are scarce. One study using the novel tank test reported that repeated exposure to ketamine (40 mg l^−1^ for 7 days, with testing 24 h later) did not alter general locomotor or anxiety-like behaviour but increased the number of full-body rotations [50]. However, this test was conducted using a front-facing camera, which may limit the accurate detection of rotational behaviour. In our study, rotations were assessed in the OTT using a top-down view, allowing for more precise measurement of body orientation. In another repeated-exposure study, locomotor activity was also unchanged after withdrawal from lysergic acid diethylamide (LSD) in adult zebrafish [60]. Our findings also differ from rodent models, where repeated ketamine exposure often results in sustained behavioural disruptions [24,61].

Zebrafish show sustained neurogenesis and regenerative ability in the central nervous system from early development through adulthood [62–65]. This may have contributed to the lack of long-term behavioural effects observed in our study, as the animals may recover from repeated exposures. Other studies have also failed to find lasting damage when ketamine was administered during early development [66]. In rodents, repeated exposure to NMDA receptor antagonists causes lasting impairment in cortical inhibitory circuits by triggering a redox-sensitive pathway. Repeated dosing increases IL-6 levels in the brain, which activates nicotinamide adenine dinucleotide phosphate (NADPH) oxidase (*Nox2*) and leads to oxidative stress that disrupts the function of parvalbumin-expressing interneurons [23,67]. These effects do not occur after a single exposure and appear only after repeated challenges. While reversible in adult rodents, they become permanent when the exposure occurs during development [67,68]. Zebrafish may be less sensitive to these changes, potentially requiring more frequent or intense exposure to produce similar long-term effects.

The NMDA receptor is highly conserved across vertebrate species, including zebrafish [45,69,70]. Subunit composition, binding affinity and overall receptor function appear sufficiently conserved to support comparable acute effects across species. Consistent with cross-species dose–behaviour comparisons, our 10–40 mg l^−1^ range falls within the behaviourally effective, sub-anaesthetic window summarized in prior comparative reviews [71]. Therefore, differences in behavioural outcomes are unlikely to result from receptor pharmacology alone. One possible explanation involves species-specific differences in ketamine metabolism. Although zebrafish express cytochrome P450 enzymes involved in drug processing [72,73], little is known about how ketamine is metabolized in this species, including how quickly it is metabolized, which metabolites are generated and whether any of them contribute to behavioural effects. Investigating ketamine's pharmacokinetics in zebrafish is important for interpreting its behavioural impact and for evaluating the model's relevance to other systems.

Our results can also inform studies on the effects of ketamine in animal models of pain, addiction and depression, by delineating sub-anaesthetic exposure ranges and context-dependent behavioural profiles. To improve the translational relevance of this model, future studies should include pharmacological validation using clinically effective antipsychotic drugs and incorporate neurochemical or physiological analyses to clarify underlying mechanisms. Expanding the behavioural assessments to cover cognitive and affective domains, alongside systematic variation in exposure conditions such as dosing schedules, developmental timing and environmental context, will allow for a more comprehensive evaluation of induced phenotypes. The absence of lasting behavioural effects may reflect the specific exposure protocols used, rather than an inherent limitation of the zebrafish model. Variations in exposure timing, duration, dose or the inclusion of environmental stressors may be necessary to produce more persistent effects.

In summary, ketamine induced clear social and locomotor changes in adult zebrafish, but the measured responses did not progressively increase across test sessions. After repeated exposure, most behavioural outcomes did not differ from controls at the 48 h assessment. By extending the predominantly acute zebrafish literature to repeated exposure and post-treatment assessment, these findings provide a more complete account of ketamine's behavioural effects. These largely negative findings contrast with reports of amplified and sustained effects in rodents, providing a basis for further investigation of ketamine responses in zebrafish and for planning future studies relevant to drug development.

## Supplementary Material



## Data Availability

All data and resources supporting this study are publicly accessible via the Open Science Framework [74]. Supplementary material is available online [75].
