# Supplementary material for: Behavioural profiling of social and locomotor responses to repeated ketamine exposure in adult zebrafish
Source: R Soc Open Sci. 2026 Nov 11;13(11):260257. doi: 10.1098/rsos.260257 (PMC13750943; doi:10.1098/rsos.260257)
Supplement: Supplementary file 1 [file rsos.260257.zip › rsos.260257/rsos260257_si_001.docx]

**Supplementary Files**

**Behavioral profiling of social and locomotor responses to repeated ketamine exposure in adult zebrafish**

Matheus Gallas-Lopes^a,c^, Daniela V. Müller^a,c^, Thailana Stahlhofer-Buss^b^, Leonardo M. Bastos^b,c^, Sofia Z. Becker^a,c^, Samara M. Bruck^a,c^, Angelo Piato^b,c^, Ana P. Herrmann^a,c^*

^a^Laboratório de Neurobiologia e Psicofarmacologia Experimental (PsychoLab), Departamento de Farmacologia, Instituto de Ciências Básicas da Saúde (ICBS), Universidade Federal do Rio Grande do Sul (UFRGS), Rua Ramiro Barcelos 2600/430, Porto Alegre, Rio Grande do Sul, 90610-264, Brazil.

^b^Laboratório de Psicofarmacologia e Comportamento (LAPCOM), Departamento de Farmacologia, Instituto de Ciências Básicas da Saúde (ICBS), Universidade Federal do Rio Grande do Sul (UFRGS), Rua Ramiro Barcelos 2600/411, Porto Alegre, RS, 90610-264, Brazil.

^c^Programa de Pós-Graduação em Farmacologia e Terapêutica, Departamento de Farmacologia, Instituto de Ciências Básicas da Saúde (ICBS), Universidade Federal do Rio Grande do Sul (UFRGS), Rua Ramiro Barcelos 2600/625, Porto Alegre, RS, 90610-264, Brazil.

*Corresponding author: Ana Paula Herrmann. Departamento de Farmacologia, Instituto de Ciências Básicas da Saúde (ICBS), Universidade Federal do Rio Grande do Sul (UFRGS), Rua Ramiro Barcelos 2600/430, Porto Alegre, RS, 90610-264, Brazil. E-mail: [ana.herrmann@ufrgs.br](mailto:ana.herrmann@ufrgs.br). Telephone: +5533082034

ORCID IDs:

Matheus Gallas-Lopes: <https://orcid.org/0000-0001-5375-2335>

Daniela V. Müller: <https://orcid.org/0000-0002-4014-1540>

Thailana Stahlhofer-Buss: <https://orcid.org/0000-0003-2183-3493>

Leonardo M. Bastos: <https://orcid.org/0000-0001-8538-6405>

Sofia Z. Becker: <https://orcid.org/0009-0008-5226-1495>

Samara M. Bruck: <https://orcid.org/0000-0002-2474-7577>

Angelo Piato: <http://orcid.org/0000-0001-5109-7306>

Ana P. Herrmann: <https://orcid.org/0000-0002-0330-0741>

**Journal:** Royal Society Open Science

**Article DOI:** 10.1098/rsos.260257

**Supplementary Fig. 1.** Additional behavioral outcomes from the social preference test in adult zebrafish exposed to different concentrations of ketamine. (A) Percent of distance traveled in the interaction zone and (C) time spent immobile, measured on days 1, 5, and 7. (B, D) Corresponding effect sizes (Cohen's d) for each outcome, with test for subgroup differences across assessment days. One-way ANOVA followed by Bonferroni post hoc test was used for A; Kruskal–Wallis test followed by Dunn's post hoc was used for C. Data are presented as mean ± SD for A, and as median with interquartile range for C. Effect sizes are expressed as Cohen's d with 95% confidence intervals. Subgroup differences tested using a random-effects model. Sample sizes: day 1 – Control = 32, Ket 10 = 32, Ket 20 = 32, Ket 40 = 32; day 5 – Control = 31, Ket 10 = 32, Ket 20 = 32, Ket 40 = 29; day 7 – Control = 31, Ket 10 = 31, Ket 20 = 31, Ket 40 = 29. Ket 10 = ketamine 10 mg/L; Ket 20 = ketamine 20 mg/L; Ket 40 = ketamine 40 mg/L.

**Supplementary Fig. 2.** Additional behavioral outcomes from the open tank test in adult zebrafish exposed to different concentrations of ketamine. (A) Percent of distance traveled in the center zone, (C) absolute turn angle, and (E) time spent immobile, measured on days 1, 5, and 7. (B, D, F) Corresponding effect sizes (Cohen's d) for each outcome, with test for subgroup differences across assessment days. One-way ANOVA followed by Bonferroni post hoc test was used for A and C; Kruskal–Wallis test followed by Dunn's post hoc was used for E. Data are presented as mean ± SD for A and C, and as median with interquartile range for E. Effect sizes are expressed as Cohen's d with 95% confidence intervals. Subgroup differences tested using a random-effects model. Sample sizes: day 1 – Control = 32, Ket 10 = 32, Ket 20 = 32, Ket 40 = 32; day 5 – Control = 31, Ket 10 = 32, Ket 20 = 32, Ket 40 = 29; day 7 – Control = 31, Ket 10 = 31, Ket 20 = 31, Ket 40 = 29. Ket 10 = ketamine 10 mg/L; Ket 20 = ketamine 20 mg/L; Ket 40 = ketamine 40 mg/L.
